# Increased 25-hydroxycholesterol as an indicator for patients with vestibular neuritis

**DOI:** 10.3389/fneur.2025.1600185

**Published:** 2025-06-20

**Authors:** Xuhua Song, Jingwei Liang, Congzhe Tian

**Affiliations:** Department of Otorhinolaryngology, Affiliated Hospital of Hebei University, Baoding, China

**Keywords:** vestibular neuritis, 25-HC, CRP, diagnosis, inflammation

## Abstract

**Background:**

Vestibular neuritis (VN) is one of the most common diseases in vestibular vertigo. 25-hydroxycholesterol (25-HC) was correlated to neuroinflammation, however, whether the level of serum 25-HC could be used to diagnose the VN occurrence remains unclear.

**Methods:**

The enrolled patients were divided into VN and healthy control groups. Afterwards, the potential risk factors were assessed in these two groups. Subsequently, the complete blood count was performed upon hospital admission.

**Results:**

The serum 25-HC and C-reactive protein (CRP) were detected in two groups using liquid chromatography-mass spectrometry and a high-sensitive immunonephelometric assay. Moreover, the correlation of 25-HC to inflammatory factors was analyzed. Finally, the receiver operating characteristic curve analysis was performed to predict the diagnosis effect of 25-HC in VN occurrence. The age, gender, BMI, living habits, disease history, and cholesterol did not affect the VN occurrence. However, 25-HC was dramatically increased in VN patients, meanwhile, peripheral blood leukocyte and neutrophil/lymphocyte ratio were also elevated in VN patients. Importantly, 25-HC was positively correlated to CRP and leukocytes. Additionally, the level of serum 25-HC could be used to predict the VN occurrence.

**Conclusion:**

Serum 25-HC may diagnose the occurrence of VN.

## Introduction

Vertigo is a series of symptoms caused by balance system dysfunction, which is a sudden illusion of self or external movement without external stimulation (1). It has been estimated that the annual prevalence of vertigo is 4.9% for the general population, and the lifetime prevalence of vertigo is about 20–30% (2–4). Vertigo is mainly divided into vestibular vertigo and non-vestibular vertigo; vestibular vertigo is further divided into vestibular peripheral vertigo and vestibular central vertigo. Perivestibular vertigo mainly includes the following common diseases: benign paroxysmal positional vertigo, vestibular neuritis (VN), Ménières disease, viral or purulent vaginitis, etc.

VN is characterized by acute, isolated, spontaneous vertigo due to unilateral vestibular differentiation, which accounts for 3.2 to 9% of the patients visiting a dizziness center (5), and has an incidence of about 3.5 per 100,000 population (6). The progression of VN can be categorized into two phases; the acute phase and the recovery phase. The acute phase usually lasts for about 14 days and during this period, the main focus of treatment is on relieving the symptoms through anti-inflammatory medication, nerve nourishment, and improving microcirculation. The recovery phase, also known as the vestibular compensation period, usually lasts for 3–6 months and includes vestibular rehabilitation training as the primary form of treatment (7). Typically, the prognosis is favorable with a low recurrence rate, but many patients may experience chronicity. Currently, the cause and pathology of VN are not well understood. Viral infections may lead to vestibular dysfunction by directly infecting neurons within the vestibular ganglia or by inducing axonal inflammation responses (8). In addition, immune-mediated mechanisms are also thought to contribute to the pathogenesis (9). Cases of vestibular neuritis have also been reported following COVID-19 infection and vaccination (10), although the underlying mechanisms remain unclear. Multiple studies support the viral etiology hypothesis of vestibular neuritis, particularly implicating herpesviruses such as herpes simplex virus type 1 and varicella-zoster virus (11). These viruses are believed to cause vestibular nerve damage either through direct neuronal invasion or secondary inflammatory processes (12).

Some studies suggest that the reactivation of neuroviruses under certain conditions may be one of the mechanisms underlying the pathogenesis of VN (12, 13). An increasing number of studies suggest that viral infections can lead to the onset and progression of VN through immune-inflammatory responses (14–16). Among the molecular mediators bridging viral infection and neuroinflammation, oxysterols—particularly 25-hydroxycholesterol (25-HC)—have emerged as key regulators due to their dual functions in antiviral immunity and immunometabolic modulation. 25-HC is an oxygenated derivative of cholesterol produced primarily by the enzyme cholesterol 25-hydroxylase, which is upregulated in macrophages and dendritic cells in response to inflammatory stimuli, particularly type I interferons (17, 18). Functionally, 25-HC acts as a key regulator of lipid metabolism by inhibiting sterol regulatory element-binding proteins, thereby suppressing cholesterol biosynthesis (19). In addition to its role in lipid homeostasis, 25-HC exhibits context-dependent immunomodulatory properties. It has been shown to suppress inflammasome activation and interleukin-1β (IL-1β) production in some settings, while promoting neuroinflammation through NLRP3 activation in others, highlighting its dual pro- and anti-inflammatory capacities (20–22).

Jang et al. (23) presented that 25-HC triggers neuroinflammation in X-linked adrenoleukodystrophy through NLRP3 inflammasome activation, which implied that 25-HC might be associated with the VN. Wu et al. (24) demonstrated that 25-HC exhibited antiviral immunity in Rhesus Macaques chronically infected with simian immunodeficiency virus and treated with antiretroviral therapy. Additionally, it has been demonstrated that 25-HC can suppress inflammation caused by IL-1 (25). Therefore, 25-HC prevents cellular cholesterol accumulation and functions as a potent modulator of neuroinflammation (26). Given the established immunomodulatory and antiviral functions of 25-HC, we hypothesized that 25-HC contributes to the inflammatory pathophysiology of VN and may serve as a diagnostic biomarker. Accordingly, this study aimed to quantify serum 25-HC levels in patients with VN and evaluate its diagnostic performance in distinguishing VN from non-vestibular vertigo.

## Methods and materials

### The diagnosis of VN patients

This is a retrospective study. The study was approved by the Ethics Committee of Affiliated Hospital of Hebei University (#HDFYLL-KY-2023-158). All 168 patients diagnosed with VN, along with 100 age- and sex-matched healthy controls, were enrolled in this study. The diagnosis of VN is based on specific criteria, which include the following: (1) the presence of acute and persistent vertigo attacks accompanied by nausea, vomiting, or unstable posture; (2) confirmation of unilateral vestibular dysfunction due to horizontal spontaneous nystagmus with rotary components directed toward the healthy ear, which can be verified via temperature and video head pulse tests; (3) the absence of hearing loss, tinnitus, or any other neurological signs; and (4) normal cerebellar and brainstem MRI images. The schematic explaining different types of vertigo and how they were categorized is shown in the Supplementary Figure S1.

### The inclusion and exclusion criteria for VN patients

To be eligible for analysis, VN patients must meet the following criteria: (1) they should have been diagnosed with VN based on the most recent criteria, after excluding other vestibular system illnesses; (2) both the blood collection time and the onset time of VN symptoms should be within 14 days (acute phase); (3) the patients should have stable vital signs, with no cognitive impairment or mental illness; (4) serum 25-HC values should be available for VN patients at the time of initial diagnosis and follow-up; (5) and the patients and their family members should be willing to participate in the study and sign an informed consent form.

To ensure an unbiased analysis, this study will exclude patients who meet the following criteria: (1) Patients with vestibular neuritis who are unwilling or unable to participate in the study or who have been lost to follow-up; (2) Patients with concurrent inflammatory diseases within the past 3 months; (3) Patients with systemic or chronic diseases, such as chronic renal or liver disease, hormonal disorders that affect 25-HC level or related conditions screened by laboratory tests and imaging examinations; (4) Patients undergoing long-term steroid therapy or osteoporosis treatment; and (5) VN patients who are lactating or pregnant.

### Biochemical indicator detection

We gathered information about the participants’ age, gender, body mass index, smoking and drinking habits, and disease history (diabetes, hypertension) along with their general health information. To analyze serum oxysterols, venous blood samples were obtained after a 10-h fasting period within 2 days of admission. The serum was separated by centrifugation and stored at −80°C until further analysis. Complete blood cell counts, total serum cholesterol, high-density lipoprotein cholesterol, low-density lipoprotein cholesterol, and cholesterol triglyceride were quantified by standard methods (27, 28).

### Quantification of serum oxysterols and CRP

The level of serum 25-HC was determined using liquid chromatography-mass spectrometry (LC–MS/MS, Thermo, MA, USA), following the previously described method (29, 30). The critical validation parameters were as follows: Limit of Detection, 1.26 fmol on-column; Average Recovery, 98.24%; CV, 5.28%. Serum CRP concentrations were measured using a high-sensitive immunonephelometric assay provided by Beckman Instruments (Indianapolis, IN, USA).

### Correlation and ROC analysis

Relationships between variables were assessed by Pearson’s correlation coefficient and Spearman correlation coefficients for parametric and nonparametric variables, respectively. To assess the classification utility of the biomarkers, area under the curve (AUC) values were computed using logistic regression models as described above and their goodness of fit was assessed using likelihood-ratio test.

### Statistical analysis

All statistical analysis was performed using SPSS software (version 26.0). The statistical data was as shown mean ± standard deviation (SD), all *p* values were shown in the table and figures. *p* with <0.05 indicated the significance of the test.

## Results

### The clinical information and biomedical indicator of VN patients

For this study, 168 patients with acute VN and 100 healthy control patients were enrolled. Analysis results showed no differences in basic information, such as age, gender, body mass index (BMI), smoking, and drinking habits, between the VN group and control group (Table 1). After collecting the serum of all patients, biomedical indicators were analyzed. The results showed no significant difference in disease history between the two groups, including diabetes and hypertension (Table 1). Finally, the biomedical indicators, such as total cholesterol (TC), triglycerides (TG), low-density lipoprotein cholesterol (LDL-C), and high-density lipoprotein cholesterol (HDL-C) have no significant difference between the two groups. It was determined that the patients in the VN group and control group had similar basic information.

**Table 1:** Clinical and laboratory characteristics of the subjects.

Parameter	VN (*n* = 168)	Control (*n* = 100)	*p*-value
Age (years)	51.20 ± 10.78	50.15 ± 7.66	0.4004
Gender			0.1773
Male (*n*)	91	52	
Female (*n*)	77	48	
BMI (kg/m^2^)	24.06 ± 2.77	23.97 ± 2.51	0.7904
Smoking [*n* (%)]	47 (27.98%)	26 (26.00%)	0.4109
Drinking [*n* (%)]	31 (18.45%)	17 (17.00%)	0.5624
Diabetes [*n* (%)]	18 (10.71%)	9 (9.00%)	0.6443
Hypertension [*n* (%)]	42 (25.00%)	22 (22.00%)	0.4355
TC (mg/dL)	221.36 ± 36.25	214.61 ± 32.44	0.1267
TG (mg/dL)	139.03 ± 34.09	131.52 ± 27.88	0.0636
LDL-C (mg/dL)	154.28 ± 36.33	150.21 ± 31.87	0.3544
HDL-C (mg/dL)	42.27 ± 5.69	41.29 ± 5.23	0.1612

VN, patients with vestibular neuritis at acute episode; BMI, body mass index; *n*, number; TC, total cholesterol; TG, Triglycerides; LDL-C, low-density lipoprotein cholesterol; HDL-C, high-density lipoprotein cholesterol. Data were as shown Mean ± SD, and *p* values were calculated via using an independent *t*-test or the chi-squared test. The differences were considered significant at *p* < 0.05.

### Serum 25-HC is increased in patients with vestibular neuritis

**Figure 1:**
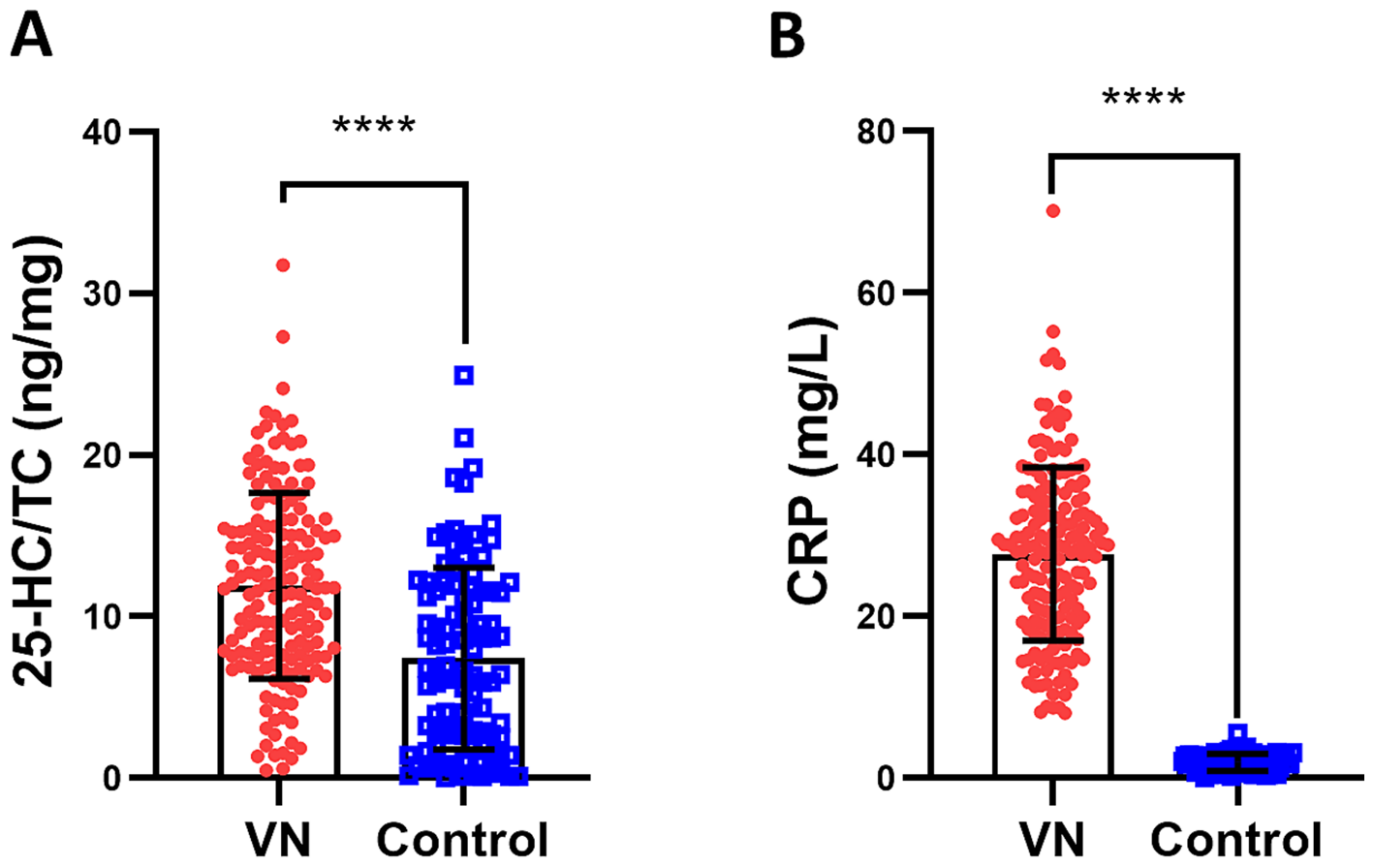
Serum 25-HC is increased in patients with vestibular neuritis. **(A)** Serum 25-HC in healthy volunteers (Control, *n* = 100) and patients with vestibular neuronitis at acute episode (VN, *n* = 168) was quantified by LC–MS/MS. 25-HC normalized to TC concentration in VN was compared with those in the controls. **(B)** The concentration of serum CRP was also measured by a high-sensitive immunonephelometric assay provided by Beckman Instruments. The data were presented as mean ± SD, *p* values were calculated via Student’s *t* test, ^****^p < 0.0001.

Currently, studies have found that 25-HC can serve as an important signaling molecule in the nervous system and participate in the occurrence of various diseases in the body (26). The levels of serum 25-HC in patients with VN and healthy control patients were measured, and the results showed that the level of serum 25-HC was higher in VN patients than in healthy patients (Figure 1A). Additionally, the level of serum CRP was also found to be increased in VN patients compared to healthy patients (Figure 1B).

### Inflammatory WBC and NLR are upregulated in patients with vestibular neuritis than those in the control

**Figure 2:**
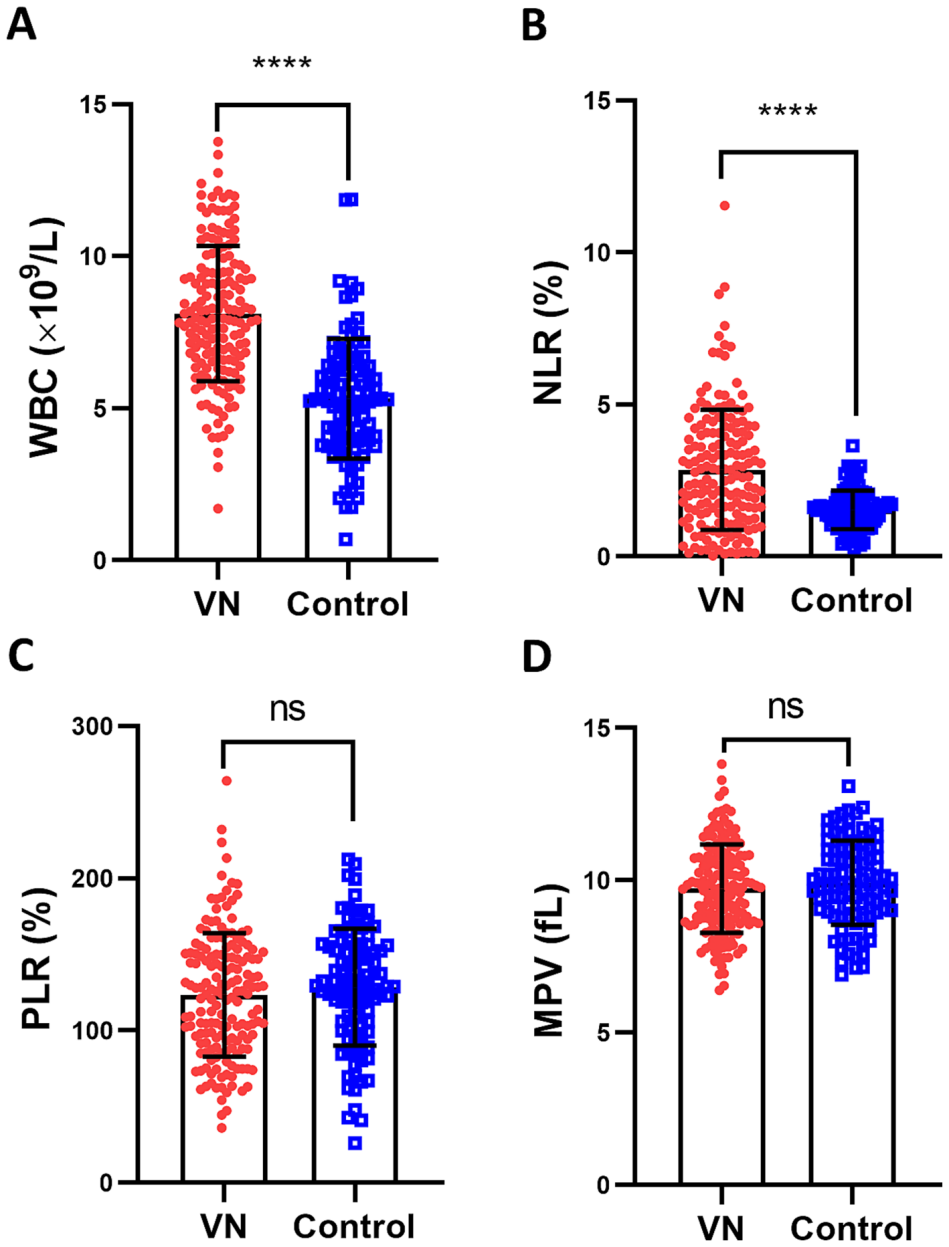
Inflammatory markers PBL and NLR are upregulated in patients with vestibular neuritis than those in the control. Complete blood count was performed upon hospital admission, including peripheral blood leukocyte (WBC, **A**), neutrophil/lymphocyte ratio (NLR, **B**), platelet/lymphocyte ratio (PLR, **C**) and average platelet volume (MPV, **D**). The data were presented as mean ± SD, *p* values were calculated via Student’s *t* test, ^****^p < 0.0001, ns means no significance.

The study aimed to investigate whether inflammation had a role in the development of VN. The researchers conducted a complete blood count test on patients with VN to measure their inflammatory indicators. The results showed that the peripheral blood leukocyte (WBC), an indicator of inflammation, was significantly higher in VN patients compared to healthy individuals (Figure 2A). Additionally, the neutrophil/lymphocyte ratio (NLR), another indicator of inflammation, was also significantly higher in VN patients (Figure 2B). However, there was no significant difference in the platelet/lymphocyte ratio (Figure 2C) and average platelet volume (Figure 2D) between VN patients and the control group. In conclusion, the findings suggested that there was an increase in inflammatory cells in patients with VN.

### 25-HC is positively correlated with inflammation

**Figure 3:**
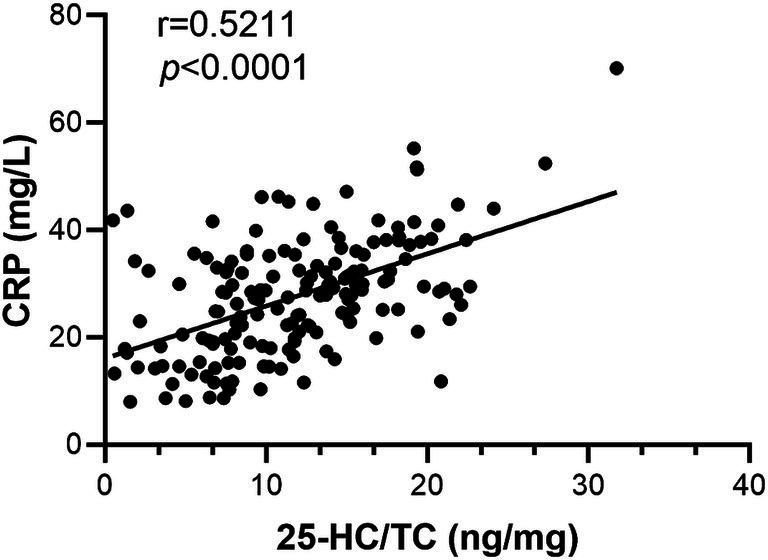
25-HC is positively correlated with CRP level in patients with vestibular neuritis. Correlation analysis between 25-HC level and CRP level in patients with VN at the acute episode, *N* = 168. Correlation analysis was calculated via Simple Linear Regression.

**Figure 4:**
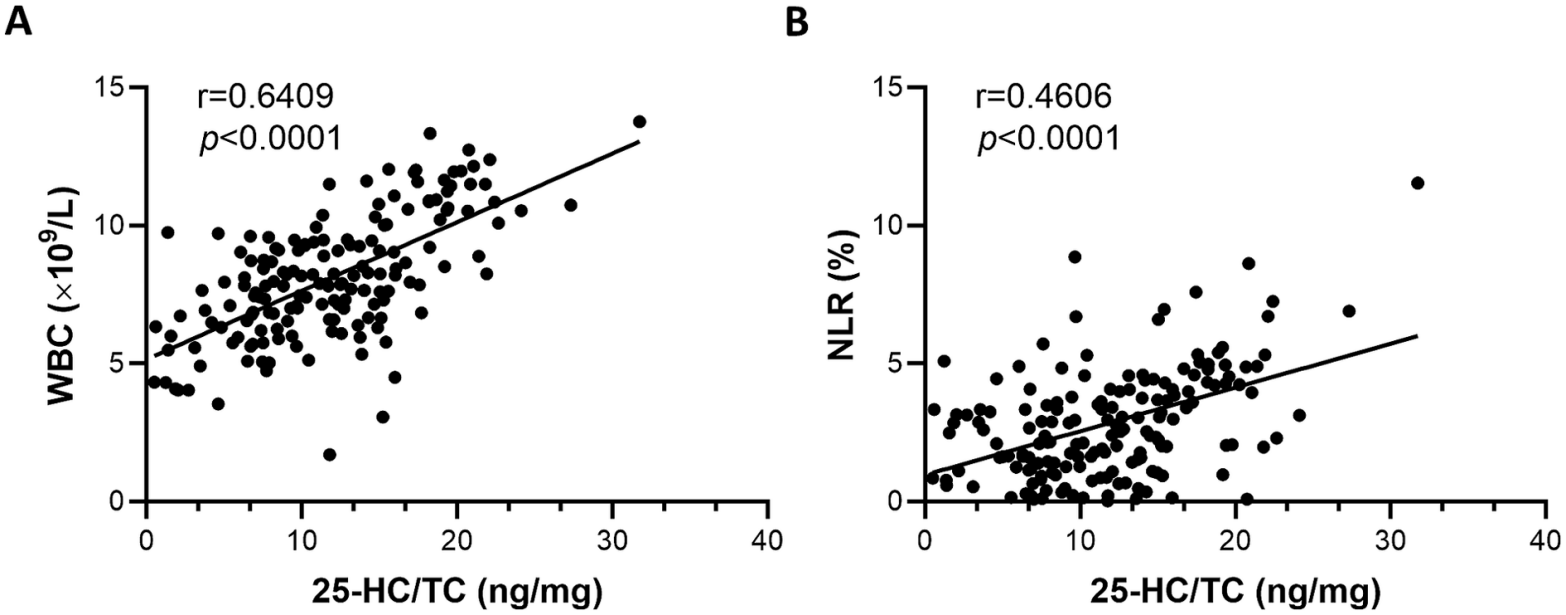
Correlation between inflammatory markers and serum 25-HC in patients with vestibular neuritis. **(A)** Correlation analysis between 25-HC level and peripheral blood leukocyte (WBC) in patients with vestibular neuronitis at the acute episode, *N* = 168. **(B)** Correlation analysis between 25-HC level and neutrophil/lymphocyte ratio (NLR) in patients with VN at the acute episode, N = 168. Correlation analyses were calculated via Simple Linear Regression.

CRP is considered as a biomarker of infection/inflammation (31), therefore the correlation of 25-HC level to CRP level was analyzed. According to the results, the level of serum 25-HC had a positive correlation with the level of serum CRP (Figure 3). This suggests that 25-HC is linked to acute inflammation. To evaluate the relationship between 25-HC and inflammation in VN more thoroughly, the correlation between the 25-HC level and various inflammatory indicators, including WBC and NLR, was analyzed. Similar to the previous findings, it was discovered that the relative 25-HC level had a positive correlation with WBC and NLR (Figures 4A,B). Our findings suggest that there is a strong correlation between 25-HC and the occurrence of VN. However, there is no significant correlation between the 25-HC level and CRP, WBC or NLR in healthy individuals (Supplementary Figure S2).

### The diagnosis value of 25-HC for VN occurrence

**Figure 5:**
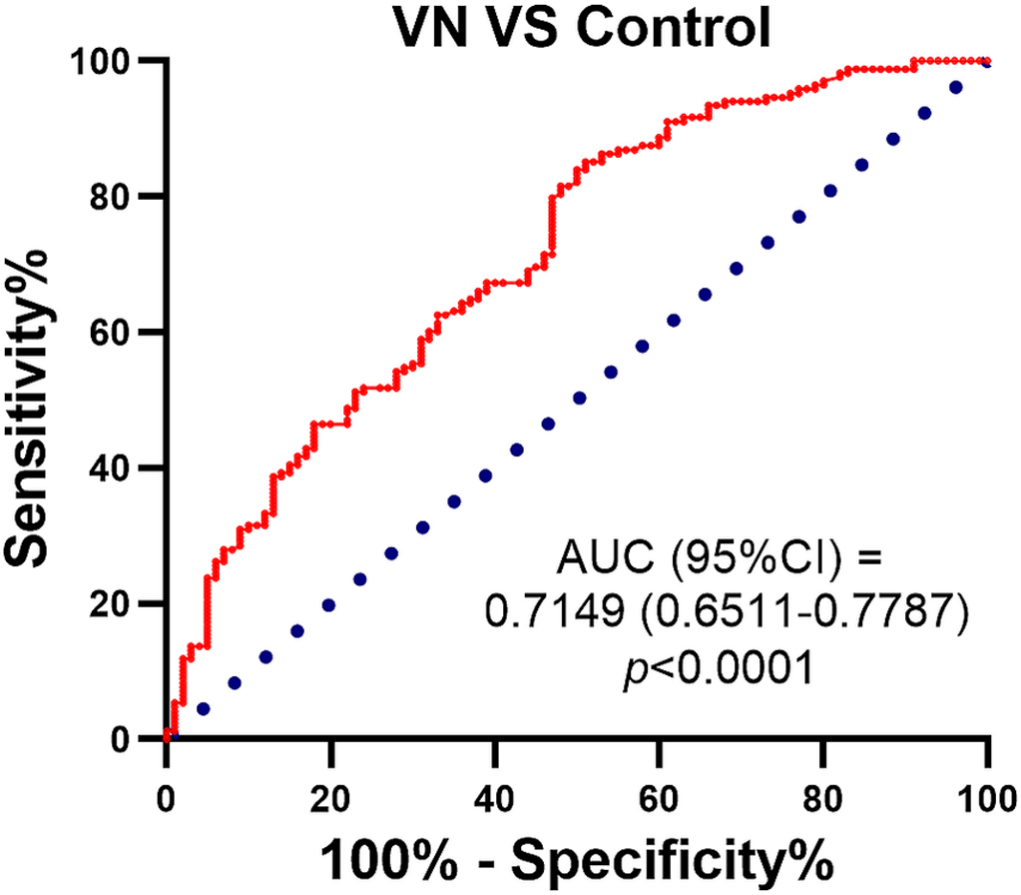
ROC curves for 25-HC levels in patients with vestibular neuritis compared with normal control. ROC curve was used to investigate the value of 25-HC in the diagnosis of patients with VN at acute episode. The predictive performance of the logistic regression model was calculated by using the area under the ROC curve (AUC), and the significance of AUC was calculated by using DeLong test. The red line represents: a model that outperforms random guessing, with a higher AUC value indicating stronger classification performance. Sensitivity and specificity: the model’s performance in distinguishing patients from controls was evaluated by using confusion matrix analysis.

A ROC analysis was performed to assess the effect of 25-HC in diagnosing patients with vestibular neuritis during the acute episode. The results revealed that the ROC curve had an AUC (Area Under the Curve) of 0.7149, indicating that 25-HC could be able to predict the occurrence of VN (Figure 5).

## Discussion

VN is a form of peripheral vertigo disease that occurs due to the loss of vestibular function caused by acute unilateral vestibular nerve conduction block (8). This condition was first defined in 1909 by Ruttin, and it is characterized by the absence of cochlear symptoms or central nervous system abnormalities (9). The exact etiology and pathophysiology of VN remain unclear. One proposed mechanism is an immune-mediated process, supported by histological findings of lymphocytic infiltration in the vestibular nerve, as reported in a rare case study by Fenton et al. (32). Additionally, MRI findings in individual case reports, such as that by Fenton et al. (32), have shown contrast enhancement of the vestibulocochlear nerve, supporting the presence of inflammation in VN (12).

All relevant risk variables were investigated in patients from the VN and control groups. The analysis results showed that age, gender, BMI, living habits, healthy condition, TC, TG, LDL-C, and HDL-C had no effect on the occurrence of VN, implying that age and gender-related changes were unrelated to VN. 25-HC is an anti-inflammatory molecule that blocks SREBP activation, regulating cholesterol biosynthesis and inflammasome activity. Moreover, 25-HC reduces inflammasome activity and subsequent IL-1β gene expression and production (25). Interestingly, interferon (IFN) activates the gene expression of cholesterol 25 hydroxylase (CH25H), thus promoting its catalytic conversion of cholesterol to 25-HC (33). Therefore, IFN stimulates the synthesis of 25-HC. This, in turn, actively suppresses signaling through the IFN receptors. There is, thus, a negative feedback loop that limits the action of IFN. Furthermore, the enzyme cholesterol 25-hydroxylase is required for the synthesis of 25-HC. This enzyme is significantly upregulated in monocytes, macrophages, and dendritic cells when they are exposed to inflammatory mediators (25, 34). These findings suggest that 25-HC may play a role in the pathogenesis of VN, a possibility that warrants further investigation.

25-HC, synthesized by CH25H, is implicated in a wide range of inflammatory diseases. In inflammatory bowel disease, although exogenous 25-HC shows limited efficacy in murine colitis models, CH25H deficiency alters disease progression, suggesting potential predictive relevance (35, 36). In lipopolysaccharide-induced acute lung injury, 25-HC levels are markedly elevated and modulate inflammatory responses (22). Notably, 25-HC is the only oxysterol consistently altered across lung inflammation models, highlighting its potential as a dynamic marker (37).

In metabolic inflammation, 25-HC is elevated in obesity and diabetes and is associated with immunomodulatory functions of adipose tissue macrophages (38). It also plays a role in neuroinflammation, as seen in Alzheimer’s disease, where increased 25-HC expression promotes proinflammatory cytokine release, implicating it as a potential biomarker (20). Furthermore, 25-HC is enriched in atherosclerotic plaques and contributes to disease progression by exacerbating endothelial dysfunction and inflammation (39). During viral infection, macrophages rapidly produce 25-HC, which triggers innate immune responses via integrin–FAK signalling (40).

Despite its broad involvement, the specificity of 25-HC as a standalone predictive biomarker remains limited. Most current studies focus on mechanistic insights, and its clinical utility may depend on co-assessment with CH25H expression and inflammatory cytokine profiles (36, 38). Additionally, 25-HC exhibits context-dependent effects—pro- or anti-inflammatory—across different disease models, warranting cautious interpretation (26, 41). Nevertheless, current evidence supports its potential as a disease-associated biomarker in specific settings such as Alzheimer’s disease, acute lung injury, and metabolic inflammation (20, 22). Given its context-dependent proinflammatory activity, the CH25H–25-HC axis has emerged as a potential therapeutic target in inflammatory disorders (42). Preclinical studies have demonstrated that genetic or pharmacological suppression of CH25H can attenuate inflammatory responses (43). Although direct evidence in vestibular neuritis is lacking, modulating this pathway may offer a novel approach to limiting vestibular nerve inflammation. Nonetheless, this therapeutic potential remains hypothetical and warrants further mechanistic and translational investigation.

A better understanding of the pharmacological behavior of 25-HC in the nervous system is critical to evaluating its mechanistic role and therapeutic relevance in VN. The blood–brain barrier serves as a selective biological membrane that tightly regulates the passage of specific molecules, notably including cholesterol, from the systemic circulation into the central nervous system. Importantly, cholesterol derivatives - which are characterized by the inclusion of hydroxyl groups within their side chains - exhibit the capacity to permeate through vascular barriers, thereby underscoring their pharmacokinetic properties (44, 45). 25-HC is a natural oxysterol that is more reactive than non-oxidized cholesterol. It is synthesized at the periphery and can enter the brain, easily diffusing through cell membranes (46). Activated microglia are responsible for producing the majority of 25-HC in the brain (47, 48). Macrophages that produce 25-HC come into contact with damaged peripheral nerves and stimulate regeneration (49). Therefore, it was supposed that 25-HC was involved in neuron inflammation which caused the VN occurrence. The evaluation of serum 25-HC level in the patients with VN showed that the level of 25-HC was significantly increased, which demonstrated that 25-HC was correlated to the VN.

CRP is a homopentameric, acute-phase, inflammatory protein that was initially discovered in 1930 (50). It is a highly conserved plasma protein that increases following inflammatory stimuli such as infection, tissue injury, and other chronic diseases. CRP is an important biomarker for detecting and monitoring systemic inflammation (51). C-reactive protein levels are known to increase dramatically in response to injury, infection, and inflammation. Produced primarily by the liver, CRP is an acute marker of inflammation (52, 53). The binding of CRP to Fc receptors results in the release of pro-inflammatory cytokines (54). There is mounting evidence suggesting CRP has a functional role in inflammation. CRP is a well-established acute marker of inflammation, with circulating levels rising during inflammatory events. In both natural and experimental conditions, CRP is deposited at sites of inflammation and tissue damage (55). CRP is a biomarker of inflammation; therefore, CRP levels were detected in patients with VN in our study. The result showed that the level of CRP was increased in VN patients, supporting the hypothesis that VN is associated with inflammation.

Due to both the 25-HC and CRP were increased in VN patients, therefore the correlation of 25-HC and CRP was analyzed and the result showed that 25-HC was positively correlated to the serum level of CRP in VN patients, which also demonstrated that 25-HC was associated with the occurrence of VN. Importantly, the peripheral blood leukocyte and neutrophil/lymphocyte ratio were significantly associated with 25-HC, which supported the evidence that 25-HC could be used to predict the occurrence of VN. Based on our findings (AUC = 0.7149), 25-HC may have potential as a supportive biomarker for VN diagnosis, although its standalone diagnostic utility is limited.

Although the AUC of 25-HC appears lower than that of CRP, its diagnostic performance may be enhanced through combination with other biomarkers, such as CRP itself, microRNAs, or proteomic indicators. Integrating multimodal data using advanced approaches like machine learning—combining biomarker profiles with clinical parameters such as video head impulse test results—could improve the differential diagnosis between vestibular neuritis and other vertigo-related disorders, including posterior circulation stroke. Future studies should explore such integrative diagnostic models to enhance clinical utility.

Although 25-HC has shown preliminary potential as a diagnostic indicator for VN, several limitations must be acknowledged. This study was conducted at a single center and lacked external validation, which may limit the generalizability of the findings. Additionally, while we demonstrated that 25-HC levels are significantly elevated in VN patients, the precise molecular mechanisms by which 25-HC contributes to VN pathogenesis were not explored. Future studies employing cellular, molecular, and animal models are needed to elucidate the mechanistic role of 25-HC in VN and to assess its translational potential. Furthermore, our analysis was based solely on clinical serum samples. Validation through experimental models, such as knockdown of CH25H (the enzyme responsible for 25-HC synthesis) in peripheral neurons, may help confirm its causal involvement in VN. Such investigations will be essential to determine whether the CH25H–25-HC axis could serve as a diagnostic or therapeutic target in VN.

## Conclusion

In summary, our findings demonstrate that serum 25-HC levels are significantly elevated in patients with VN and are positively correlated with key inflammatory markers including CRP, WBC, and NLR. These results suggest that 25-HC is closely associated with the inflammatory response in VN. Moreover, ROC analysis indicates that 25-HC has moderate diagnostic value for identifying VN during the acute phase, highlighting its potential as a biomarker for VN occurrence.

## Data Availability

The original contributions presented in the study are included in the article/Supplementary material, further inquiries can be directed to the corresponding author.
