# Supplementary material for: Increased 25-hydroxycholesterol as an indicator for patients with vestibular neuritis
Source: Front Neurol. 2025 Jun 20;16:1600185. doi: 10.3389/fneur.2025.1600185 (PMC12226294; doi:10.3389/fneur.2025.1600185)
Supplement: Supplementary file 1 [file Table_1.DOCX]

**Supplementary materials**

**Figure S1. Different types of vertigo.** The schematic explaining different types of vertigo and how they are categorized.

**Figure S2. Correlation between inflammatory markers and serum 25-HC in healthy individuals. A.** Correlation analysis between 25-HC level and CRP level in healthy individuals. N=100. **B.** Correlation analysis between 25-HC level and peripheral blood leukocyte (WBC) in healthy individuals. N=100. **C.** Correlation analysis between 25-HC level and neutrophil/lymphocyte ratio (NLR) in healthy individuals. N=100.
